# causal effects of air pollution on dementia: a mendelian randomized study

**DOI:** 10.1002/alz.090084

**Published:** 2025-01-09

**Authors:** YUNYUN GUO, Shireen Sindi, Rui Wang, Lefkos T Middleton, Miia Kivipelto

**Affiliations:** ^1^ The Ageing Epidemiology (AGE) Research Unit, School of Public Health, Imperial College London, London, United Kingdom, London United Kingdom; ^2^ Karolinska Institutet, Department of Neurobiology, Care Sciences and Society, Division of Clinical Geriatrics, Center for Alzheimer Research, Stockholm Sweden; ^3^ The Ageing Epidemiology (AGE) Research Unit, School of Public Health, Imperial College London, London United Kingdom; ^4^ Department of Neurobiology, Care Sciences and Society, Division of Clinical Geriatrics, Center for Alzheimer Research, Karolinska Institutet, Stockholm/Solna Sweden

## Abstract

**Background:**

The 2017 Lancet Commission on Dementia Prevention, Intervention and Care included air pollution in the list of potential risk factors for dementia. Air pollution has been associated with an increased risk of dementia in observational studies, but the causal relationship remains unclear. In this study, we used a two‐sample Mendelian randomisation (MR) analysis to assess the causal relationship between PM_2.5_, PM_10_, NO_2_ and NO_X_ exposure and the occurrence of dementia, including Alzheimer’s disease (AD), vascular dementia, Lewy body dementia, and frontotemporal dementia.

**Method:**

We extracted 5 single nucleotide polymorphisms associated with PM_2.5_, 22 single nucleotide polymorphisms associated with PM_10_, 6 single nucleotide polymorphisms associated with NO_2_ and 8 single nucleotide polymorphisms associated with NO_X_ as instrumental variables from a genome‐wide association study based primarily on European ancestry. Summary‐level statistics were obtained from the Dementia GWAS database and the FinnGen consortium. MR estimation was based on the inverse variance weighting (IVW) method as the primary method, supplemented by the MR‐Egger and weighted median (WM) methods. Finally, multiple validity tests and sensitivity analyses were performed to obtain robust and valid estimates.

**Result:**

According to the IVW method, we found a positive association between PM_10_ and AD risk (OR: 2.03, 95% CI: 1.06‐ 3.89, p = 0.03); NO_2_ may increase the risk of AD (OR: 14.41, 95% CI: 10.95‐ 18.97, p = 0.04). However, there is insufficient evidence that PM_2.5_ and NO_X_ are associated with an increased risk of future dementia.

**Conclusion:**

Our study suggests that PM_10_ and NO_2_ are causally associated with AD risk. PM_10_ and NO_2_ may increase the risk of AD in people of European origin. More animal experiments or population‐based observational studies with long follow‐up are needed in the future to explore the intrinsic mechanisms of PM_10_, NO_2_ and AD.

